# Anterior cervical surgery to treat diffuse idiopathic skeletal hypertrophic combined with cervical disc herniation: A case report

**DOI:** 10.1097/MD.0000000000026097

**Published:** 2021-06-04

**Authors:** Kun Gao, Yafei Cao, Weidong Liu, Shufen Sun, Yihong Wu, Weiji Yu

**Affiliations:** Shenzhen Traditional Chinese Medicine Hospital, Shenzhen, China.

**Keywords:** cervical spine, disc herniation, DISH

## Abstract

**Rationale:**

Diffuse idiopathic skeletal hyperostosis (DISH) is a skeletal disease characterized by calcification of anterolateral ligaments of the spine and the rest of the body. DISH combined with disc herniation induces complex symptoms and is more difficult to treat. Here, we describe a complicated case of a patient diagnosed with DISH as well as cervical disc herniation that was successfully treated using anterior cervical surgery.

**Patients concern:**

A 69-year-old Asian male experienced dysphagia and weakness in his left limbs. He also experienced a stiff neck and right slant over a 6-month period.

**Diagnosis:**

An X-ray revealed calcification of the C4-7 vertebral front edge and a narrowed intervertebral space between C5/6. Coronal and sagittal computerized tomography (CT) and magnetic resonance imaging (MRI) both showed compression of the spinal cord at the cervical 5/6. Esophagography revealed that osteophytes in the anterior margin of vertebral body (C4-C7) oppressed the esophagus.

**Interventions:**

An operation focused on anterior cervical C5/6 disc fusion and anterior vertebral C4-7 osteophyte removal was performed.

**Outcomes:**

After the operation, X-ray and CT showed that most osteophytes were removed and spinal cord compression was relieved. One day following the operation, both dysphagia and numbness in limbs were improved. Strong recovery was observed at the three-month follow-up exam.

**Lessons:**

This complex DISH combined with disc herniation case is rarely observed in patients. Anterior cervical disc fusion and anterior vertebral osteophyte removal were effective treatment measures. This case study provides insight into treating cases presented with cervical spine complications associated with DISH combined with other ailments.

## 1 Introduction

Diffuse idiopathic skeletal hyperostosis (DISH), formally described by Forestier and Rotes-Querol in the 1950s, is a skeletal disease characterized by calcification of the anterolateral ligaments of the spine and calcification of ligaments in other areas of the body. This disorder is more common in middle-aged and elderly individuals, with the incidence rates being 25% and 15% for males and females over the age of 50, respectively.^[1]^ The exact pathophysiology behind this disease is unknown. Many scholars believe it may be related to degeneration, genetics, metabolism or endocrine, and toxin factors.^[2]^

Cervical DISH causes dysphagia, hoarseness, stiffness, and pain in the back of the neck. It can also cause snoring and expiratory dyspnea.^[3]^ About 50% of patients diagnosed with DISH show ossification of the posterior longitudinal ligament of the cervical spine,^[4]^ which can cause clinical manifestations of spinal cord compression. In this report, we present a rare case of a patient suffering from DISH combined with a cervical disc herniation. The patient showed symptoms of dysphagia, torticollis, and limb numbness. Our treatment using careful anterior cervical surgery to remove osteophytes and fuse centrum was successful. This study was approved by the EC office of Shenzhen Traditional Chinese Medicine hospital and patient rights were protected. The patient was informed of his rights and he signed an informed consent form.

## 2 Case report

### 2.1 Clinical presentation

A 69-year-old Asian male patient arrived at our hospital due to a stiff neck and slant to the right. He also experienced dysphagia and weakness in his left limbs but did not experience dizziness, a headache, panic, or tightness in the chest.

He had a history of coronary atherosclerotic heart disease for 5 years and was taking medication regularly to treat this. He did not have a history of hypertension, diabetes, or nephropathy, nor did he have a history of hepatitis, tuberculosis, or other infectious diseases. Lipoma resection was performed on the left face 3 years prior and appendectomy was performed over 40 years prior. No trauma, blood transfusions, poisoning, or and penicillin anaphylaxis were involved in this case. The patient did not drink regularly and never smoked.

On examination, cervical movements were limited. Both the left transverse process and articular process of the cervical spine were tender. Muscle tension in the limbs was normal and there was no obvious atrophy or disorder in fine motor ability. Muscle strength of the main muscles of the left limb were slightly decreased and the right main muscles were normal (left biceps brachi 4/5, triceps brachii 4/5, dorsal forearm 4/5, volar forearm 4/5, hand grasp 4/5); (right biceps brachi 5/5, triceps brachii 5/5, dorsal forearm 5/5, volar forearm 5/5, hand grasp 5/5); (left quadriceps femoris 4/5, tibialis anterior 4/5, gastrocsoleus 4/5, flexor longus 4/5); (right quadriceps femoris 5/5, tibialis anterior 5/5, gastrocsoleus 5/5, flexor longus 5/5). Bilateral Eaten signs (−), Spurling signs (−), Hoffman signs (+), Babinski signs (−), patellar clonus (−), ankle clonus (−). Bilateral knee tendon and Achilles tendon reflexes were both normal.

Laboratory data revealed high creatinine (118 μmol/L) and uric acid (528 μmol/L) levels.

### 2.2 Radiologic evaluation

**Figure 1:**
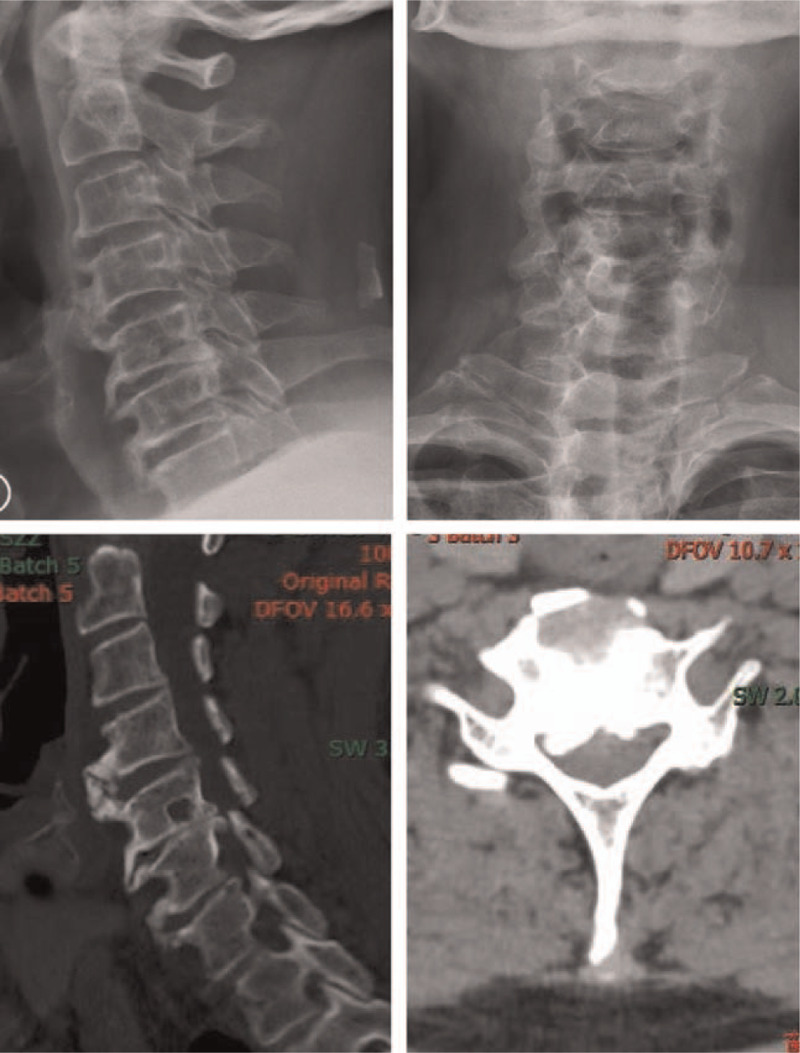
Preoperative imaging of the cervical spine. A and B, The X-ray examination showed C4-7 anterior vertebral osteophyte, nuchal ligament calcification, and the intervertebral space of C5/6 was narrowed. C and D, Axial and sagittal CT showed C4-7 anterior vertebral osteophyte and a calcification in the C5/6 spinal canal.

**Figure 2:**
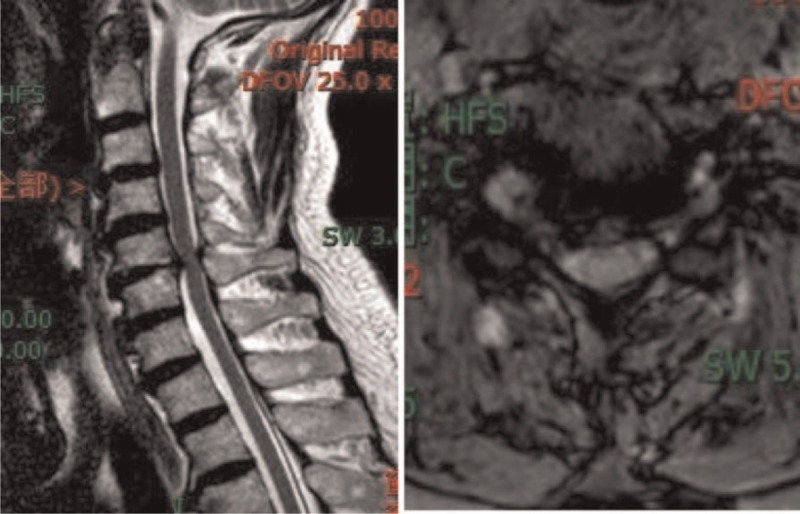
Axial (A) and sagittal (B) MRI showed C5/6 spinal cord compression. MRI = magnetic resonance imaging.

**Figure 3:**
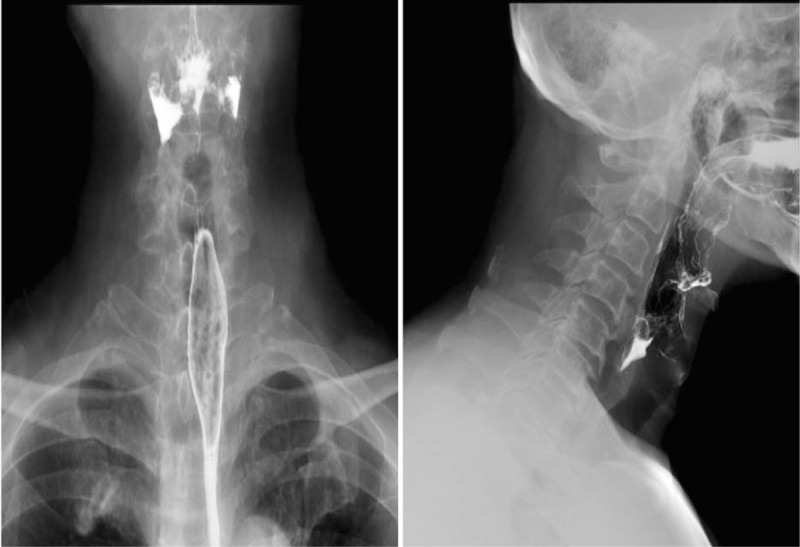
A and B, The esophagography showed the anterior edge of C4-7 esophagus was compressed.

An X-ray showed degeneration of cervical vertebra bone, calcification of the nuchal ligament, and narrowing of the intervertebral space between C5/6 (Fig. 1 A and B). Coronal and sagittal computerized tomography (CT) showed herniation of the cervical 3/4 and cervical 5/6 discs (central type); a cervical 4/5, 6/7 disc bulge; cervical bone hyperplasia and nuchal ligament calcification (Fig. 1 C and D). Both X-ray and CT scans showed that the anterior margin of cervical vertebrae (C4-C7) contained extensive flow calcification. Magnetic resonance imaging showed cervical disc herniation (cervical 3/4, 4/5, 5/6), cervical 5/6 level spinal cord compression, cervical 5/6 vertebral endplate inflammation, and C5 vertebral hemangioma (Fig. 2 A and B). Esophagography showed osteophytes present in the anterior margin of vertebral body (C4-C7) that were oppressing the esophagus (Fig. 3 A and B).

### 2.3 Treatment

An anterior cervical surgery was performed on the patient. During the operation, a transverse slot about 4 cm in length was generated in the left anterior part of the neck. First, the C5/6 intervertebral space was identified and a discectomy and zero-p interbody fusion were performed. The C4-7 vertebrae were located and osteophytes in front of the vertebral body were removed using bone biting forceps.

### 2.4 Histopathology

Isolated osteophytes were sent for pathological examination. Hematoxylin-eosin staining showed that the specimen contained bone tissue, degenerated ligaments, and a small amount of fibrocartilage tissue with extensive calcification.

### 2.5 Outcomes and follow-up

**Figure 4:**
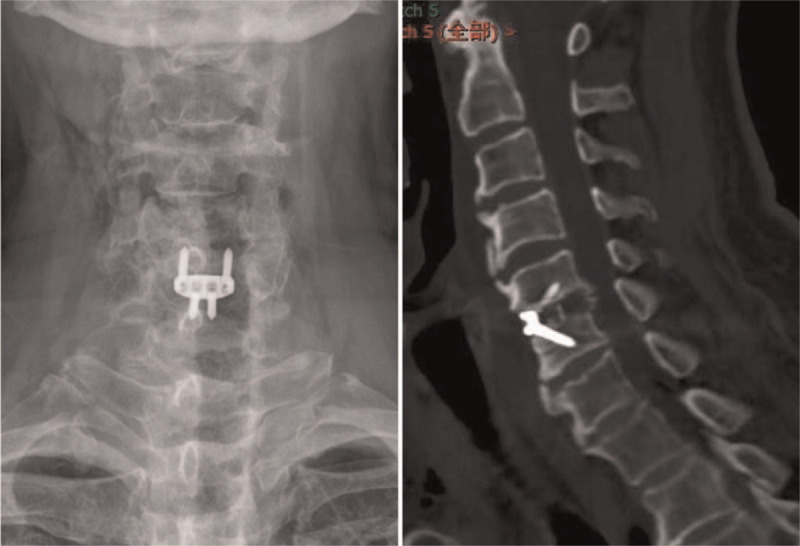
Postoperative imaging of the cervical spine. X-ray (A) and CT (B) showed most of the osteophytes in C4/5 and C6/7 were removed and the fusion cage was in good position. CT = coronal and sagittal computerized tomography.

After surgery, X-ray and CT showed that most osteophytes in C4/5 and C6/7 were removed and that the fusion cage was in an ideal position (Fig. 4 A and B). One day following the operation, the patient was able to get out of bed with a neck brace. Dysphagia was improved and limb numbness was relieved. Three months later, the patient was not experiencing dysphagia or limb numbness. On physical examination, cervical movements were not limited. Muscle strength of the main muscles of the left limb was norma. Bilateral Eaten signs (−), Spurling signs (−), Hoffman signs (−), Babinski signs (−), patellar clonus (−), ankle clonus (−). Bilateral knee tendon and Achilles tendon reflexes were both normal.

## 3 Discussion

The symptoms presented in this case were complicated, including dysphagia, neck stiffness, and limb numbness. Numbness in the left lower limb is especially rare. After surgical treatment, dysphagia, upper limb numbness, and lower limb numbness were all relieved. This is the first case where this has been reported.

Thus far, the etiology of DISH is not clear. Histology revealed that ossification was initially seen in the adjacent tissue in front of the vertebral body.^[5]^ Focal calcification and ossification were observed with use of a microscope in the anterior longitudinal ligament and occasionally new bone was formed by the cartilage island in the ligament. There were irregular ossifications and osteophyte formation in the attachment of the anterior longitudinal ligament to the vertebral body. In addition the levels of osteophytes gradually increased.^[6]^

Dysphagia is the main symptom associated with DISH and is related to the following factors: direct compression by osteophytes, limited activity of pharyngeal structures such as the epiglottis cartilage, serrated stenosis of the pharynx causing enlargement of the proximal piriform fossa and food residue, and inflammatory edema of the esophagus which can cause fibrous tissue hyperplasia and lead to endogenous stenosis of the esophagus.^[7]^ Therefore, esophageal barium meal examination should be performed before an operation to determine the site of stenosis. An otolaryngologist should be consulted to determine whether there is esophageal disease caused on its own.^[8]^ Simple osteophyte resection can relieve symptoms in patients experiencing dysphagia.^[9,10]^ In addition, follow-up visits show a low recurrence rate. If combined with vertebral segment instability or compression of the spinal cord, an additional intervertebral disc extraction and interbody fusion would be performed.

In the case presented here, cervical CT and barium meal examination showed obvious C4-C7 osteophyte hyperplasia causing serious compression of the esophagus. Therefore, dysphagia was mainly caused by C4-C7. In addition, the patient presented limb numbness, neck stiffness, and torticollis deformities. Cervical magnetic resonance imaging and CT showed C5/6 disc herniation and spinal cord compression. Thus, we decided to perform an anterior cervical C5/6 disc fusion and anterior vertebral C4-7 osteophyte removal operation. After the operation, symptoms of esophageal compression and limb numbness were significantly improved. To reduce the incidence of complications, preoperative preparation and preoperative evaluation should be improved. Approximately 10% of patients experienced vocal cord paralysis, stroke, Horner syndrome, infections, esophageal leakage, and other serious complications.^[11]^

It is important to mention that dysphagia is also a common complication of anterior cervical surgery. Tracheoesophageal traction training before anterior cervical surgery improves the compliance of the trachea and esophagus and improves the occurrence of dysphagia after an operation.

However, osteophytes in front of the cervical vertebra were also close to the back of the esophageal wall in the case presented here. Improper pushing of the anterior cervical vertebra may cause rupture of the esophagus. Tracheoesophageal traction training may not be suitable for patients with cervical DISH. Preoperative guidance should be given to patients with tracheoesophageal traction training based on the size, location, direction, and thickness of osteophytes in the esophageal wall

Abdel-Aziz et al indicated that patients with EAT-10 scores up to 20 were subjected to conservative treatment (e.g., diet modifications, nonsteroidal anti-inflammatory drugs, corticosteroids, muscle relaxants, and ant reflux medications), whereas patients with EAT-10 scores greater than 20 were subjected to osteophyte resection.^[9,10]^ However, since patients with severe or very severe dysphagia showed significantly smaller improvement, some scholars suggested that early intervention guarantees a higher success rate.^[11–13]^

In conclusion, this study demonstrates a complex case of cervical disease caused by DISH combined with cervical disc herniation. Anterior cervical fusion and anterior vertebral osteophyte removal showed a strong therapeutic effect. Short-term effects of this treatment were significant and long-term effects need to be observed in the future. This case study will aid in treatment strategies for cervical spine complications caused by DISH as well as other maladies.

## Author contributions

**Conceptualization:** Yafei Cao.

**Data curation:** Kun Gao, Weidong Liu, Shufen Sun, Yihong Wu, Weiji Yu.

**Formal analysis:** Kun Gao.

**Funding acquisition:** Yafei Cao.

**Investigation:** Weidong Liu, Shufen Sun.

**Project administration:** Weidong Liu.

**Visualization:** Weiji Yu.

**Writing – original draft:** Kun Gao.

**Writing – review & editing:** Yafei Cao.
